# Silk fibroin-based embolic agent for transhepatic artery embolization with multiple therapeutic potentials

**DOI:** 10.1186/s12951-023-02032-9

**Published:** 2023-08-19

**Authors:** Linlin Shi, Danni Li, Qianqian Tong, Guorong Jia, Xiaohong Li, Lan Zhang, Qingqing Han, Rou Li, Changjing Zuo, Wei Zhang, Xiao Li

**Affiliations:** 1 Zhejiang Hospital of Integrated Traditional Chinese and Western Medicine, Hangzhou, 310005 Zhejiang China; 2 Department of Nuclear Medicine, Shanghai Changhai Hospital, Shanghai, 200433 China; 3 Shanghai Institute of Applied Physics, Chinese Academy of Sciences, Shanghai, 201800 China; 4 Department of Interventional Radiology, Zhongshan Hospital, Fudan University, Shanghai, 200032 China

**Keywords:** Silk fibroin, Embolic agents, Iodination, Transhepatic artery embolization, Biomedical behaviors, Drug loading, Multiple therapeutic potentials, Enzymatic degradation, Clearance, Liver cancer

## Abstract

**Background:**

The excellent physicochemical and biomedical properties make silk fibroin (SF) suitable for the development of biomedical materials. In this research, the silk fibroin microspheres (SFMS) were customized in two size ranges, and then carried gold nanoparticles or doxorubicin to evaluate the performance of drug loading and releasing. Embolization efficiency was evaluated in rat caudal artery and rabbit auricular artery, and the *in vivo* distribution of iodinated SFMS (^125^I/^131^I-SFMS) after embolization of rat hepatic artery was dynamically recorded by SPECT. Transhepatic arterial radioembolization (TARE) with ^131^I-SFMS was performed on rat models with liver cancer. The whole procedure of selective internal radiation was recorded with SPECT/CT, and the therapeutic effects were evaluated with ^18^ F-FDG PET/CT. Lastly, the enzymatic degradation was recorded and followed with the evaluation of particle size on clearance of sub-micron silk fibroin.

**Results:**

SFMS were of smooth surface and regular shape with pervasive pores on the surface and inside the microspheres, and of suitable size range for TAE. Drug-loading functionalized SFMS with chemotherapy or radio-sensitization, and the enhanced therapeutic effects were proved in treating HUH-7 cells as lasting doxorubicin release or more lethal radiation. For artery embolization, SFMS effectively blocked the blood supply; when ^131^I-SFMS serving as the embolic agent, the good labeling stability and embolization performance guaranteed the favorable therapeutic effects in treating *in situ* liver tumor. At the 5th day post TARE with 37 MBq/3 mg ^131^I-SFMS per mice, tumor activity was quickly inhibited to a comparable glucose metabolism level with surrounding normal liver. More importantly, for the fragments of biodegradable SFMS, smaller sized SF (< 800 nm) metabolized in gastrointestinal tract and excreted by the urinary system, while SF (> 800 nm) entered the liver within 72 h for further metabolism.

**Conclusion:**

The feasibility of SFMS as degradable TARE agent for liver cancer was primarily proved as providing multiple therapeutic potentials.

**Graphical abstract:**

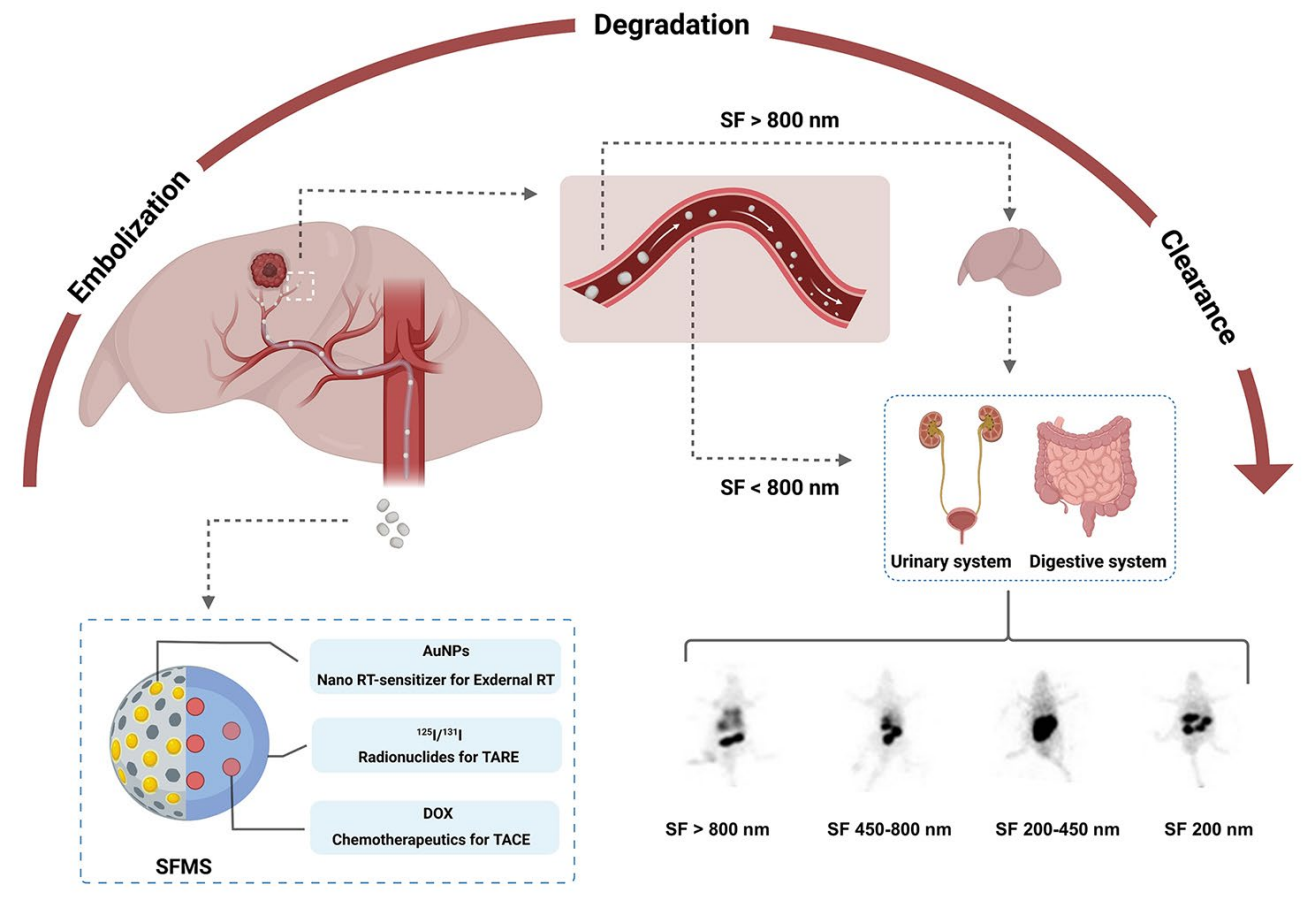

**Supplementary Information:**

The online version contains supplementary material available at 10.1186/s12951-023-02032-9.

## Introduction

There are about 600,000 new cases of liver cancer in the world every year [1]. Due to the rapid deterioration, the potential liver dysfunction and often late diagnosis, only about 30% of patients are eligible to receive radical treatments, such as surgical excision and percutaneous ablation. Therefore, palliative treatment offers an alternative for patients with advanced liver cancer, especially in inoperable cases. Transhepatic artery embolization (TAE) has become an adjuvant strategy for the clinical treatment of liver cancer due to its minimally invasive nature and high drug utilization rate [2]. Because liver tumors are predominantly supplied with blood by the hepatic artery, while the surrounding normal liver tissue is primarily supplied by the portal vein system, TAE strategy can occlude the target blood vessel to block the supply of nutrients at the tumor site, and then deliver high concentration of drugs or high doses of radiation to the local tumor, enabling the treatment of primary liver cancer or liver metastasis. Additionally, due to the enhanced efficacy of various synergistic treatment modes, agents with single embolic function are no longer sufficient to meet clinical needs, and multifunctional embolic agents have gradually shown significant advantages in clinical application. Besides the embolization, the therapeutic efficacy of multifunctional embolic agents can be improved by strengthening the drug loading and degradation performance.

Microspheres are one kind of the most commonly used solid embolic materials in clinical practice, and characterized as structured, not easy to aggregate, and feasible for functional modification [3, 4]. According to the degradation properties, microspheres can be divided into degradable ones, such as human serum albumin (HSA) microsphere, starch microsphere, etc. [5, 6], and non-degradable ones, such as polyvinyl alcohol (PVA) microsphere, gelatin microspheres, and so on [7, 8]. After degradation of biodegradable microspheres, vascular recanalization provides a pathway for repeatable tumor embolization with less damage to the body even after the rare ectopic embolism. Therefore, more biodegradable materials are actively researched and developed. For example, biodegradable silk fibroin (SF) shows excellent potential in clinical application research. As an easily available natural bio-polymer that are extracted from silkworm cocoons, SF has a long history being used as surgical suture, which mainly attributed to the fact that SF and its degradation products are non-toxic to cells and the body and will not or rarely cause inflammation or immune rejection[9, 10]. Owing to its unique mechanical properties, diverse processability, good histocompatibility and controllable biodegradability, SF has been promoted as tissue engineering materials such as bone tissue scaffolds[11], a wound healing dressing and drug sustained-release carriers to control the delivery and release of drugs[12–15].

At present, the biomedical applications of SF mainly focused in external uses such as prosthetic skin, suture and so on [9, 16]. Different from *in vitro* or on the surface, the local dosage of silk fibroin microspheres (SFMS) used as an embolic agent is larger, usually reaching more than 10 mg level, and directly interacts with arterial blood supply. Therefore, the study on SFMS properties such as drug loading, arterial embolization, degradation and distribution of fragments *in vivo* are warranted. In this study, SFMS were used to load gold nanoparticles (AuNPs) or doxorubicin (DOX) to evaluate the ability to support functional nano-materials or chemotherapeutic drugs. Furthermore, a systematic and visible research protocol with radioactive iodinated SFMS was designed to verify SFMS for TARE. The embolization performance and size-dependent pattern of *in vivo* biological clearance were evaluated via dynamic and timed single photon emission computed tomography (SPECT) so as to explore the biomedical behaviors of SFMS. Hopefully, this work may pave the way for utilizing silk fibroin-based embolic agents and provide a reference for precise biomedical application of SF agents.

## Materials and methods

### Preparation of SF-based embolic agent

#### Characterization of SFMS

SFMS (< 5 μm or > 20 μm) that were synthesized via self-assembly of purified SF were customized by Suzhou SIMATECH Biotechnology Co., LTD. For morphology evaluation, scanning electron microscopy (SEM, S-4800, Hitachi company, Japan) was used to observe the surface, pore distribution and internal structure of SFMS with the magnification between 100 and 3000 times. The pictures of SFMS captured by SEM was imported into Image J software, and 100 microspheres were randomly selected to measure the diameters, so as to evaluate the average particle size and the size distribution of microspheres.

An amount of SFMS were mixed and ground with potassium bromide. The microspheres were characterized with Fourier transform infrared spectrometer to acquire the infrared spectra with characteristic wavelength range of 600–4000 cm^-1^, so as to evaluate the potentials of functional modification.

#### DOX loading of SFMS as a TACE agent

1 mg DOX was loaded to 50 mg SFMS via mixing and vibrating at 37 ℃ for one hour. The microspheres suspension was centrifuged to collect the DOX-loaded SFMS. The loading efficiency was quantified via UV measurement at 254 nm of super solution before and after mixing.

#### AuNPs loading of SFMS as a radiotherapy sensitizer

50 mg SFMS were suspended in 1 mL AuNPs solution (2 mg/mL), rotated and oscillated for 30 min, 80 r/min. The mixture was centrifuged at a low speed for 3 min at 300 r/min. The centrifuged SF-AuNPs (10 µL) and washed filtrate were separately treated with freshly prepared aqua regia for 2 h, quantitatively analyzed the Au element by ICP-MS to determine the loading rate of AuNPs. AuNPs-loaded SFMS was observed with TEM to verify the inter distribution of nanoparticles.

#### Radioactive Iodine labeling to SFMS and the corresponding stability

10 mg SFMS were suspended in 0.01 M PBS, rotated and oscillated for 30 min to form 10 mg/mL suspension. 3.5 µL sodium iodide (Na^131^I, 37 MBq/µL) solution and 50 µL SFMS suspension were added into Iodogen tube successively. At room temperature, Iodogen tube was placed on vortex mixer for 20 min to obtain ^131^I-labeled SFMS suspension. Labeling capacity was determined via labeling 3.7, 37, 370, 3700 MBq to 100 µg SFMS, and the labeling rate > 95% was defined as meeting the requirements of utter iodination.

Whatman chromatographic strip paper was used as stationary phase and normal saline as mobile phase. The developed chromatographic filter paper was taken out and dried. The radio-fraction was analyzed by radio-thin layer chromatography. The origin of the sample was ^131^I-SF and the distal end was ^131^I which was not successfully labeled. In vitro stability test was performed as the radiochemical purity of labeled products was determined after they were mixed with 0.01 M PBS at 37 ℃ and incubated for 4 h, 24 and 72 h. In vitro stability test was performed as the radiochemical purity of labeled products that was determined after they were equivolumically mixed with 0.01 M PBS or 5% Bovine Serum Albumin at 37 ℃ and incubated for 4 h, 24 and 72 h.

SFMS (< 5 μm) were labeled with iodine-125 (^125^I) by the above method. Wistar rats were injected with 100 µL ^125^I-SF suspension containing about 37 MBq/10 mg via tail vein. At different time points (1–72 h) after injection, SPECT/CT (Symbia T16, Siemens, Germany) was used to perform whole body scans in rats to observe thyroid iodine uptake. The acquisition of images was based on the following parameters: low-energy high-resolution collimator imaging, acquisition matrix: 128 × 128, energy peak: 35 keV, window width: 20%; frame number: 60 s/frame. CT scanning, tube voltage: 130 kV, tube current: 30 mA, layer spacing: 1 mm; reconstruction thickness: 1 mm.

#### Inhibition of cellular viability with drug-loaded SFMS

For DOX-loaded SFMS, 5 × 10^4^ HUH-7 (human hepatocellular carcinoma) cells were co-incubated with 1 mg DOX-loaded SFMS or SFMS respectively for 15 h. To verify the continuous inhibition resulted from the controlled release of DOX, the medium of another group with 1 mg DOX-loaded SFMS was replaced with normal medium at 6 h after the beginning of co-incubation. Cellular viability of above three groups was measured with CCK-8 kit and compared with control group of no treatment.

For AuNP-loaded SFMS, 5 × 10^4^ HUH-7 cells were co-incubated with 0.2 mg SFMS, SFMS-AuNPs, ^131^I-SFMS or ^131^I-SFMS-AuNPs respectively for 24 or 48 h. The initial radiation of I-131 was set as 3.7 MBq/well. Cellular viability of above four groups was measured with CCK-8 kit and compared with control group of non-treatment.

### Application of SFMS as embolic agent

#### Animals and raising

Male Wistar rats (8–10 weeks of age, weighing 180–200 g) and male New Zealand white rabbits (9–12 weeks of age, weighing 2.0-2.5 kg) were purchased from Shanghai Sippr-BK LAB Animal Co., LTD. Animal care and all experimental procedures were performed under the approval of the Ethics Committee of Shanghai Changhai Hospital.

#### Artery embolization using SFMS

##### Embolization of rat caudal artery

Male Wistar rats were anesthetized with intraperitoneal injection of 3% pentobarbital sodium (0.1 mL/kg). The middle caudal artery of rat was punctured at the proximal end of the middle and upper 1/3 of the rat tail. After the needle was pulled out, the guide wire catheter was quickly sent through the puncture point into the vessel for about 1 cm, then the guide wire was withdrawn and 400 µL SFMS suspension (> 20 μm, 15 mg/mL) was injected through the catheter.

##### Embolization of rabbit auricular artery

Male New Zealand white rabbits were placed in a rabbit fixator. 300 µL SFMS suspension (> 20 μm, 35 mg/mL) was slowly injected into the central auricular artery of rabbits, and the gross changes in embolized ears were inspected and photographed at 0, 7th and 21th day post injection.

##### Embolization of hepatic artery

Rats were anesthetized with anesthesia machine using an isoflurane-oxygen mixture (2-2.5% isoflurane, 500–700 mL/min). A ventral midline incision was made to expose the liver, the common hepatic artery branch was further exposed to distinguish with the proper hepatic artery and the gastroduodenal artery. The gastroduodenal artery was ligated with suture in front of the distal part of the right gastric artery, and the common hepatic artery was clamped with artery clip. The guide wire catheter was inserted into gastroduodenal artery and sent to the beginning of the branch of proper hepatic artery, then the guide wire was withdrawn. After normal saline was injected to confirm no leakage, 200–400 µL ^125^I-SFMS (37 MBq/30 mg) suspension was slowly injected through the catheter. The catheter was slowly exited and the artery of proximal gastroduodenum was ligated. The wound was closed and disinfected.

Whole-body scans were performed on SPECT/CT at different time points (5 h and20 h) after hepatic artery embolization in rats. Image acquisition parameters were the same as 2.1.4. The distribution of ^125^I-SFMS in liver and extrahepatic organs was recorded.

#### Artery embolization using ^131^I-SFMS

##### Rat hepatocellular carcinoma model

N1S1 (rat hepatocellular carcinoma) cells were cultured in the medium of 90% DMEM plus 10% fetal bovine serum in a constant temperature incubator at 37 ℃ and 5% CO_2_. N1S1 cells suspension (containing approximately 5 × 10^6^ cells) in exponential growth period were mixed with equal volume Matrigel and placed on ice for reserve.

Rats were anesthetized with anesthesia machine with gas flow at 500–700 mL/min with the isoflurane concentration at 2-2.5%. After the left lobe of liver was exposed, 1 mL syringe was inserted into the liver at the oblique above the middle of left lobe, and 100 µL cell matrix glue mixture was slowly injected. After the mixture solidified, the needle was removed and the cotton swab gently pressed the injection site to stop bleeding. Intramuscular injection of penicillin sodium (200,000 U/mouse) and dexamethasone (2.5 mg/mouse) was performed every three days post operation to prevent infection and induce immunosuppression.

The successful preparation of animal model was verified by ^18^ F-FDG PET/CT at 7th day after liver transplantation of tumor cells. Rats with tumor xenografts of 0.3–0.8 cm in maximum diameter and increased ^18^ F-FDG uptake, but without any indications of systemic tumor metastasis or infection, were enrolled in the study. The maximum tumor diameter and maximum standardized uptake value (SUV_max_) of the enrolled rats were recorded.

##### ^131^I-SFMS TARE

The hepatocellular carcinoma rats were injected with 200–400 µL ^131^I-SFMS suspension (37 MBq/3 mg) into the proper hepatic artery of rats through catheter. The puncture and operation methods were the same as those in the above part. After embolization, SPECT/CT scan was performed at 0 h, 3 h, 12 h, 20 h, 40 h, 60 h, 110 h, 160 h to monitor the retention and distribution of embolized microspheres at the tumor site. Image acquisition was based on the following parameters: high energy collimator imaging, acquisition matrix: 128 × 128; energy peak: 364 keV; window width: 20%. CT scan, tube voltage: 130 kV; tube current: 35 mA; thickness of reconstructed layer: 1 mm.

After attenuation correction, the tumor, normal liver, gastrointestinal tract, muscle and other areas of interest were mapped, and the radioactive counts were measured. The unit radioactivity count of the left lower limb thigh muscle was taken as the background, and the result was expressed as the ratio of tumor, liver and gastrointestinal radioactivity count to background. At the same time, taking the initial radiation count in the tumor area as the initial background, the ratio of cumulative radiation count of the tumor to background at each scanning time point was calculated, and the curve of radiation dose ratio change in the tumor area was drawn. The attenuation correction was performed on the curve according to the physical half-life of ^131^I. The internal dose of absorbed radiation was assessed utilizing the phantom of “300 g Rat” following the MIRD method [17]. The semi-quantitative information of pharmacokinetic parameters were adopted for the assessment of radiation dosage of embolized lesions and total body.

At 5th day of TARE, ^18^ F-FDG PET/CT scan was performed. Tumor volume and SUV_max_ were measured. The change rate of SUV_max_ was calculated as: [(SUV_max_pre_ - SUV_max_post_)/SUV_max_pre_] x 100%. SUV_max_ of peripheral normal liver was measured as control.

### Enzymatic degradation

SFMS (> 20 μm) in 0.01 M PBS was degraded at 37 ℃ in dark conditions for 24 h by the enzymatic action of elastinase (1 mg/mL). The samples were obtained at 6 h and 24 h, and SEM was used to analyze the surface morphology and structure of the SFMS fragments.

### Metabolism of SFMS-fragment

#### Filtration and classification of SF

Lyophilized SF powder (< 2 μm) was bought from Meilun Biotechnology Company. Accordingly, a certain amount of SF powder was suspended in water and oscillated with ultrasound for 10 min. The obtained SF suspension was subsequently filtered by 0.8 μm, 0.45 μm, and 0.2 μm syringe filter in order to separate the particles into four size rages: > 800 nm (abbreviated SF_800_), 450–800 nm (abbreviated SF_450-800_), 200–450 nm (abbreviated SF_200-450_) and < 200 nm (abbreviated SF_200_).

#### Acquisition of time-dependent biodistribution

*In vivo* biodistribution study was carried out on Wistar rats, which were randomly divided into four groups (three rats for each group). The four iodine-125 labeled SF suspensions (0.5 mL ^125^I-SF, containing 8.3 MBq of ^125^I) with different particle sizes was injected into four groups of rats via tail vein, respectively. Four groups of rats were scanned by SPECT/CT coherently in dynamic mode immediately after injection, and followed by static mode at different time points. Specifically, the whole-body images were consecutively collected every 30 s for the first 10 min, and every 1 min from 10 to 40 min. Then all rats were then scanned at 1 h, 3 h, 6 h, 10 h, 24 h, 48 and 72 h, respectively.

### Quantification and statistics

Attenuation correction of I-131 was performed in quantification of embolization efficiency. The differences, such as the changes of SUV_max_ of ^18^ F-FDG PET, were evaluated with paired *t*-test. A difference with p-value less than 0.05 was significant.

## Results

### Drug loading of SFMS

#### Characterization of SFMS

**Fig. 1:**
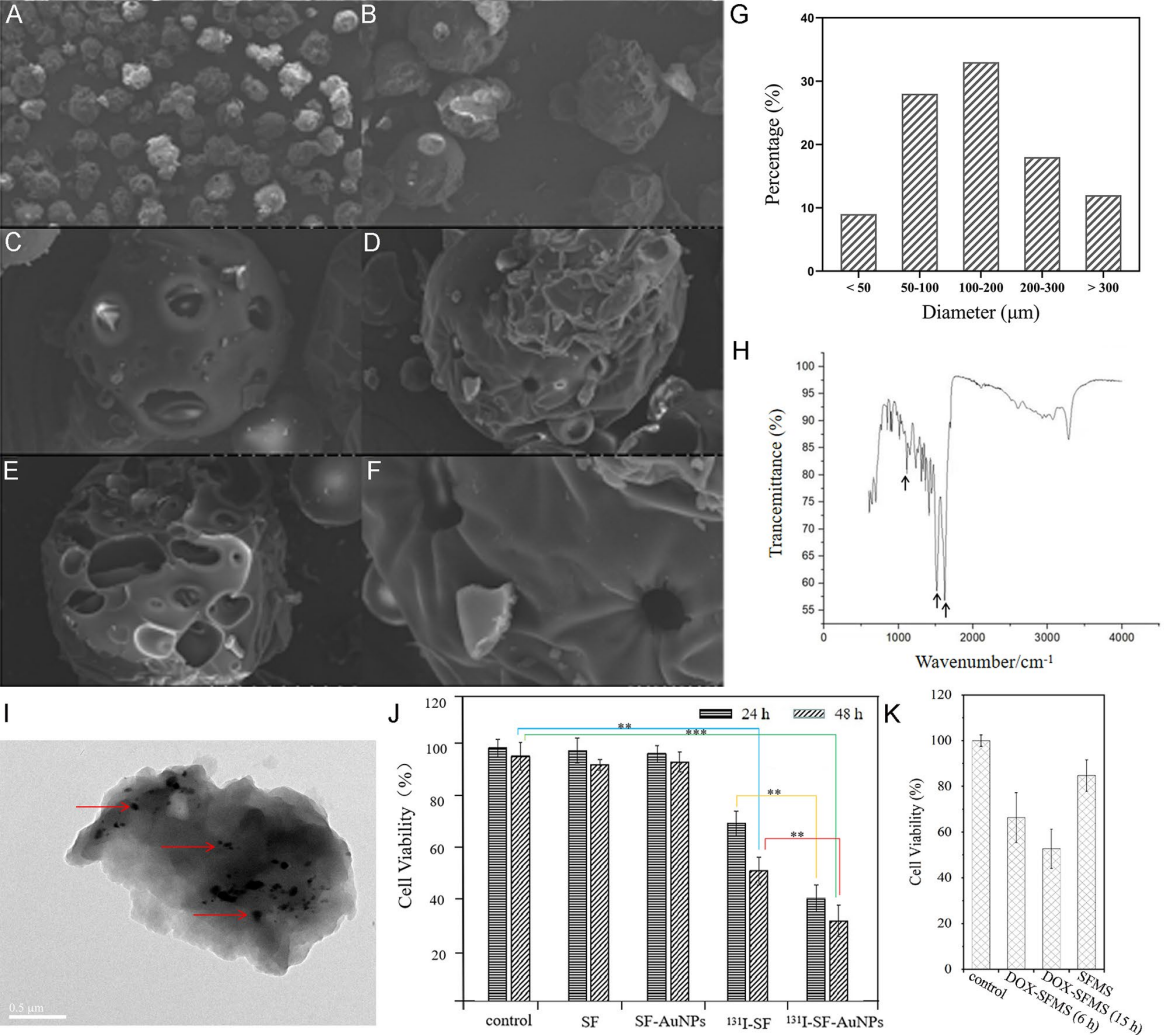
The morphology of silk fibroin microspheres characterized by SEM, including amplification factor × 100 (**A**), amplification factor × 300 (**B**), amplification factor × 1000 (**C**), amplification factor × 1000 (**D**), cross section with amplification factor × 1000 (**E**), amplification factor × 3000 (**F**); size distribution of SFMS (> 20 μm) (**G**); IR spectra of SFMS (**H**); TEM of AuNPs-loaded SFMS (< 5 μm) with arrows pointing to AuNPs (**I**); Viability of HUH-7 cells incubated with SF, SF-AuNPs, ^131^I-SF and ^131^I-SF-AuNPs for 24 and 48 h (**J**); The viability of HUH-7 cells treated with SFMS, DOX-SFMS for 6 and 15 h (**K**)

As shown in Fig. 1A-F, SEM revealed that the surface of dry SFMS was of smooth and regular shape, with no obvious mutual adhesion between the microspheres. The surface of SFMS contained holes with diameters ranging from sub-micron to micron, and a large number of spherical holes existed in the internal cross section of the microspheres.

SFMS (> 20 μm) was of size distribution as: 9% with particle size less than 50 μm, 28% of 50–100 μm, 33% of 100–200 μm, 18% of 200–300 μm, and 12% of more than 300 μm (Fig. 1G). The average particle size was 151.23 ± 11.57 μm, which was consistent with the commonly used size range of liver cancer embolization microspheres.

As shown in Fig. 1H, IR spectrum was of the absorption peak at 1650 cm^-1^, the amide I peak of SF; the absorption peak at 1515 cm^-1^, the amide II peak of SF; and the absorption peak at 1239 cm^-1^, the amide III peak of SF. The abundant surface amino groups of the SFMS were beneficial to functional modification.

#### AuNPs and DOX loading

Based on the ICP-MS results, the average load of AuNPs in 1 mL (50 mg) AuNPs-SFMS suspension was 0.59 ± 0.25 mg, and AuNPs loading rate was 29.50 ± 0.67%. AuNPs distributed equally in the SFMS as shown in TEM of AuNPs-loaded SFMS (Fig. 1I). AuNPs-SFMS was biosafe to HUH-7 cell, but AuNPs effectively sensitized the I-131-based RT. For the I-131-induced decrease of cell viability, AuNPs further enhanced the inhibiting effects on proliferation of tumor cells (Fig. 1J). In detail, after 24 h co-incubation, the cell viability of ^131^I-SF group (64.57 ± 2.39%) and ^131^I-SF-AuNPs group (37.89 ± 1.93%) was lower than that of control group (93.68 ± 2.29%), showing obvious cytotoxicity (p < 0.05). In addition, ^131^I-SF-AuNPs group showed greater cytotoxicity than the ^131^I-SF group, and the difference was statistically significant (*p* < 0.05), indicating that AuNPs sensitized the radiation damage of ^131^I.

The average load of DOX in 1 mL (50 mg) DOX-SFMS suspension was 0.98 ± 0.02 mg with a nearly complete drug load. Cellular viability manifested that the controlled release of DOX guaranteed the persistent inhibition on cellular proliferation. Due to the continuous releasing of DOX to medium, the group of 15 h co-incubation with DOX-SFMS was of a significantly decreased cellular viability than that of 6 h co-incubation (52.7 ± 8.6% vs. 66.3 ± 10.9%) (Fig. 1K).

#### Radioactive Iodine labeling

**Fig. 2:**
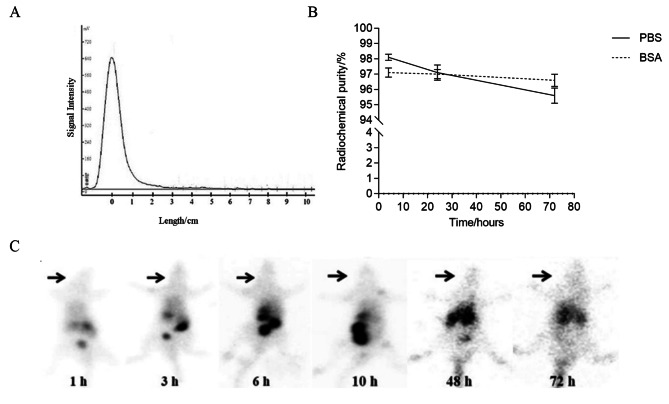
TLC spectrum of ^125^I-SF (**A**) and in vitro stability in 0.01 M PBS and 5%BSA (**B**); SPECT images at different time points after ^125^I-SF intravenously injected into Wistar rats (**C**), and arrows pointing to the thyroid area

This study employed two types of radioactive iodine, I-131 and I-125, for different purposes. I-131 was employed in the treatment of liver cancer via TARE, while I-125 was used as a tracer to monitor the SF and its degradation products. The radiolabeling yield of ^125^I-SF prepared by Iodogen method was more than 99%. When the labeling rate > 95%, the labeling capacity of I-131 to SFMS was more than 370 MBq/mg. After purification of labeling system of 370 MBq and 100 µg SFMS, the labeling capacity was 1924 ± 210 MBq/mg. Typical TLC spectrum was shown in Fig. 2A. The R_f_ value of ^125^I-SF was 0, and that of free ^125^I was 0.8-1. At 37 ℃, the radiochemical purity of ^125^I-SF were 98.1 ± 0.2%, 97.1 ± 0.5% and 95.6 ± 0.5% in 0.01 M PBS for 4 h, 24 and 72 h, respectively. Accordingly, the radiochemical purity of ^125^I-SF was 97.1 ± 0.3%, 97.0 ± 0.3% and 96.6 ± 0.4% in 5%BSA for 4 h, 24 and 72 h, respectively (Fig. 2B).

After ^125^I-SF was injected into rats through the tail vein, the physiological characteristics of rats did not change significantly. SPECT images showed varying degrees of distribution of ^125^I-SF in parts of the rat’s organs at different time points after injection (Fig. 2C). There was no concentration in the thyroid area until 72 h resulting from in vivo radioactive iodine de-labeling. In a certain period of time, the SFMS labeled by the above method showed no obvious de-iodination *in vivo*.

### TAE Application of SFMS

#### Artery embolization of rat caudal artery and rabbit auricular artery

**Fig. 3:**
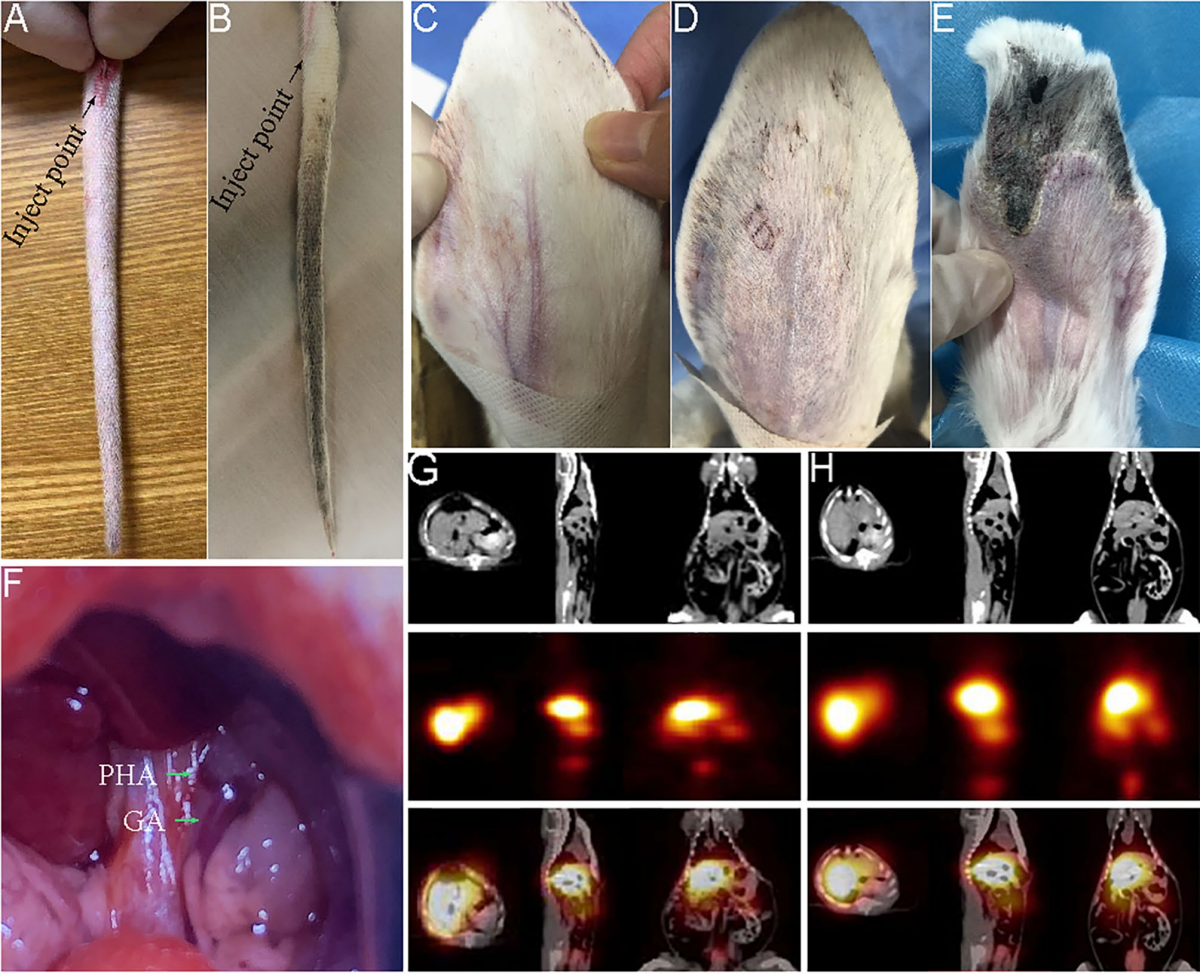
Embolization efficiency on normal arteries. Changes of rat tail after embolization by caudal artery catheterization (**A** for pre-injection and **B** for 7th day post injection); Changes of rabbit ear after central artery embolization (**C** for immediate post injection, **D** for 7th day post injection, **E** for 21st day post injection); Diagram of the rat’s proper hepatic artery (**F**) and SPECT image after ^125^I-SF embolization via the rat’s proper hepatic artery (**G** for 5 h, **H** for 20 h). PHA: proper hepatic artery; GA: gastroduodenal artery

The caudal artery of the rat showed no significant resistance during intubation, and there was no local congestion or edema in the tail of rat after embolization with SFMS (Fig. 3A). On the 7th day, blood supply from the proximal end of puncture site to the distal end of rat tail had interrupted, resulting in necrosis, skin blackening and tissue stiffening (Fig. 3B). Ultimately, the necrotic rat tail was broken on the 10th day.

SFMS was smoothly injected into the rabbit central auricular artery without subcutaneous eminence. After artery embolization, the color of blood vessels changed from a bright red to a light red and eventually to white, with no obvious congestion (Fig. 3C). On the 7th day, there was no recanalization of distal end, and the rabbit ear showed slight edema and inflammation (Fig. 3D). On the 21st day, the central vessel near the puncture site showed the normal blood supply, exhibiting bright red color. The proximal rabbit ear was soft, while the distal tissue was necrotic and blackened, with the epidermis appearing atrophied and hardened, and the terminal artery occlusion disappeared (Fig. 3E).

As a result, SFMS was proven to be an effective embolization agent for the rabbit auricular artery and rat caudal artery, with no obvious toxic effect on non-embolized tissue.

#### Artery embolization of normal rat hepatic artery

Figure 3 F shown the localization of the visible proper hepatic artery of rat pre-embolization. Systemic SPECT/CT (5, 20 h) images of rats after transhepatic artery embolization with ^125^I-SF suspension were shown in Fig. 3G H. After hepatic arterial injection, SPECT images showed that ^125^I-SF was still concentrated in the whole liver at 20 h, suggesting that SF as an embolic agent was not completely embolized in the main trunk of the proper hepatic artery, and ^125^I-SF was also embolized in the branches and peripheral vessels of the liver. A small amount of radioactivity was detected in the gastrointestinal tract and bladder, which was the normal distribution of SF with small particle size after entering the blood circulation from the embolization site.

#### Therapeutic effects of ^131^I-SFMS as a TARE agent

**Fig. 4:**
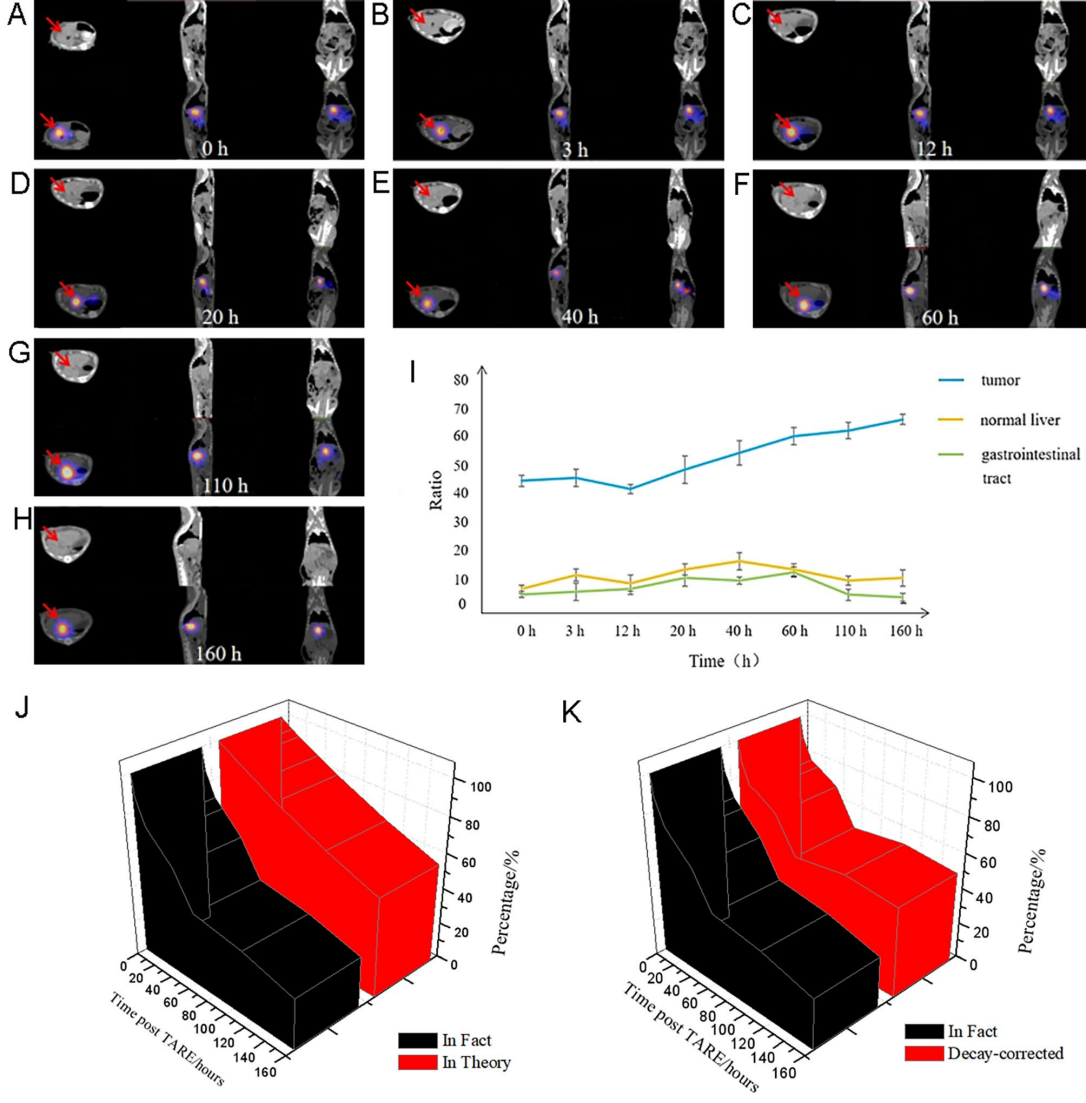
SPECT/CT images (**A-H**) of rat hepatocellular carcinoma within 160 h after ^131^I-SFMS TARE, with arrow pointing to tumor; Dynamic changes in the ratios of embolized focus, peripheral liver and intestinal tract to background (**I**); Cumulative effect of internal exposure post ^131^I-SFMS TARE (**J-K**).

Rats with hepatocellular carcinoma was injected with ^131^I-SFMS through the proper hepatic artery. Immediately after embolization (0 h), SPECT/CT images showed a high concentration of ^131^I-SFMS in the tumor area, and no significant concentration was observed in the rest of the body. SPECT/CT images at each time point after embolization were shown in Fig. 4A-H. From immediately after embolization to 160 h, the tumor area maintained high radioactivity, while no significant ^131^I-SFMS was observed in other non-embolized organs. In addition, there was no iodine uptake in the thyroid at 160 h, indicating the good *in vivo* stability of ^131^I-SFMS.

The changes in the count ratios of tumor, normal liver and gastrointestinal tract to muscle (background) were shown in Fig. 4I. The cumulative radiation in the tumor area was about 50–70 times higher than the background, and the ratio of tumor to background showed a gradually increasing trend. The radiation counts of normal liver and gastrointestinal tract near the tumor were about 10 times of background and gradually decreased after reaching the maximum accumulation. Due to the physical decay of I-131, although the radiation counts in the tumor area and the whole body decreased, the tumor area still maintained a higher radioactivity compared to the whole body. This higher ratio of tumor to normal tissue favors of the sharper imaging and the maintenance of a higher therapeutic radiation dose in the tumor area. In addition, radioactive deposits in normal tissues were efficiently cleared, minimizing radiation damage to normal tissues. When set the initial injection dose in tumor as the baseline (100%), the unit radioactive deposition in normal liver decreased from 21.0% at 3 h to 4.6% at 160 h; similarly, in the gastrointestinal tract, it decreased from 11.4% at 3 h to 1.8% at 160 h.

The radioactivity count ratio and corrected curves for the tumor area were shown in Fig. 4I and K. The area under the curve represented the cumulative radiation dose. According to the first correction method (Fig. 4J), 100% embolization of SFMS was assumed at the tumor site, and the maximum theoretical cumulative dose was obtained based on the half-life of I-131. The actual measurement realized about 61.74% of the maximum theoretical cumulative dose, suggesting that there was room for improvement in the embolization performance of SFMS. According to the second correction method (Fig. 4K), under the existing embolization performance of SFMS, the radioactivity count of tumor area at different time points was corrected according to the half-life of I-131. According to the metabolic characteristics of embolic agent, the cumulative radiation dose within 160 h was about 74.14% of the actual maximum cumulative dose. Under the existing embolization performance, the time required to reach 90% of the maximum value is about 228 h, i.e., 1.18 half-lives of I-131. Based on the semi-quantitative information of pharmacokinetics, the estimated accumulative dosage in lesions was as high as 264 Gy in the first 160 h, and the estimated accumulative dosage in “total body” was less than 12 Gy meantime, after taking into account a combination of factors such as drug residues in the catheter.

**Fig. 5:**
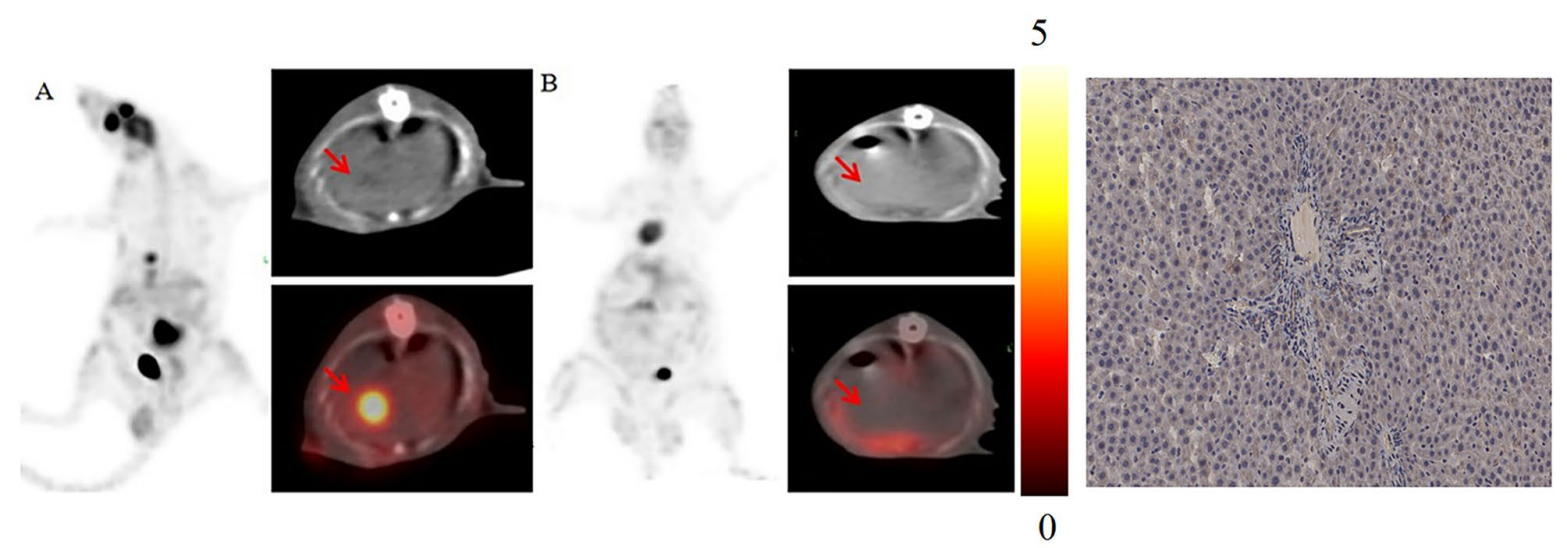
^18^ F-FDG PET/CT images of rat models with hepatoma before (**A**) and after (**B**) ^131^I-SFMS TARE, with arrow pointing to the tumor area., and corresponding IHC (40 ×) on HIF-1α at 14 d post-TARE.

After 5 days of ^131^I-SFMS embolization, ^18^ F-FDG PET/CT images (Fig. 5) showed no obvious radioactive uptake in the original site of the tumor of liver, and no low-density lesion was observed on the CT image. The SUV_max_ of the tumor area were 3.66 ± 0.98 pre-treatment and 0.45 ± 0.17 post-treatment. The change rate in SUV_max_ of tumor pre- and post-treatment was 87.70 ± 2.71%. The SUV_max_ of surrounding nontumoral liver tissue was 0.65 ± 0.11, which was slightly higher than that of tumors post treatment, but not statistically significant. CT images showed no obvious signs of necrosis in the surrounding nontumoral liver tissue. The above results evidenced the feasibility of the ^131^I-SFMS in radiotherapy embolization, as well as biosafety for surrounding tissues.

### Enzymatic degradation in vitro and following clearance

#### Morphology of degraded fragments

**Fig. 6:**
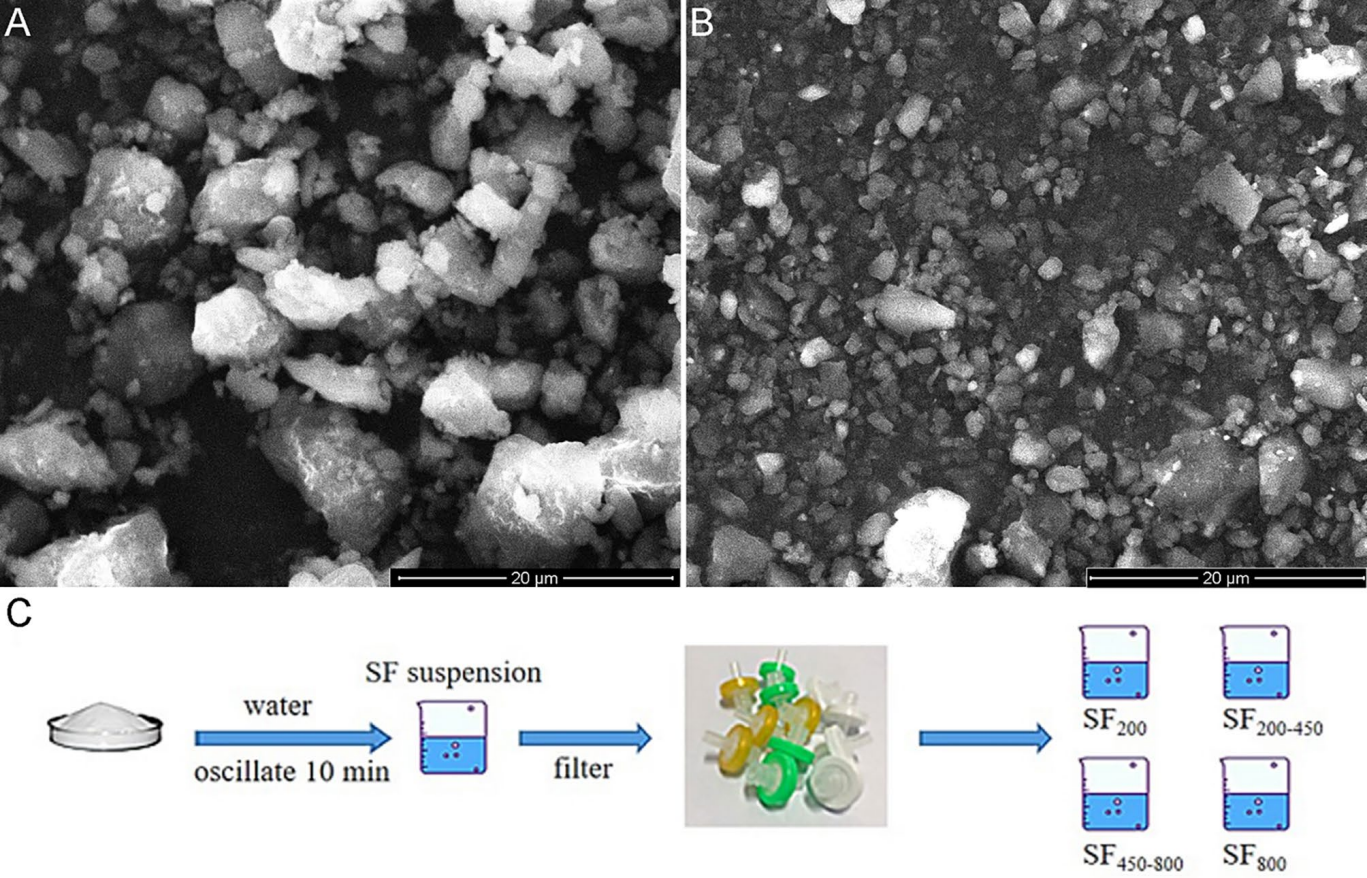
SEM of SFMS after enzymatic degradation for 6 h (**A**) and 24 h (**B**); and the procedure of classification via diameters (**C**)

SEM images of SFMS after enzymatic degradation for 6 and 24 h were shown in Fig. 6A and B, respectively. SEM images showed that the SFMS after enzymatic degradation had an amorphous structure and a wide range of sizes.

#### Super early in vivo distribution

**Fig. 7:**
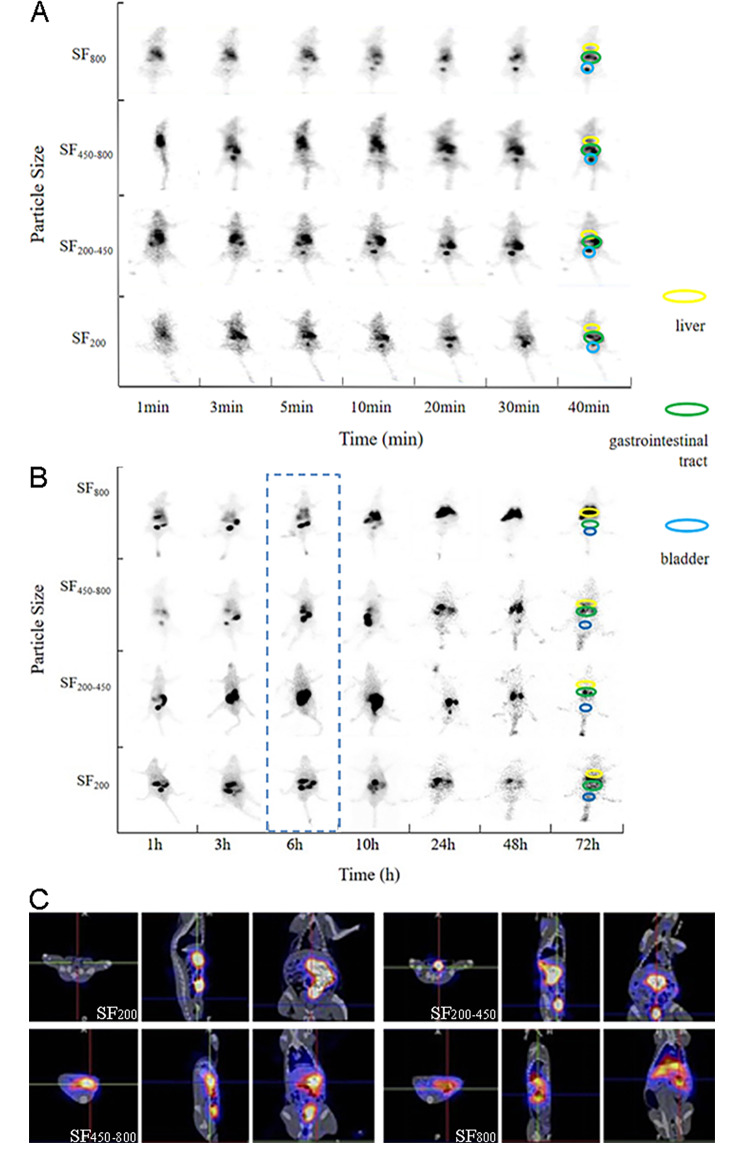
SPECT images of rats after injecting ^125^I-SF via tail vein within 72 h. (**A**) planar images of the early 40 min; (**B**) planar images of rats within 72 h; (**C**) SPECT/CT images 6 h after injection

The typical *in vivo* distribution of SF in the early 40 min was shown in Fig. 7A. The early distribution patterns of SF with four sizes were similar. After injection, ^125^I-SF was mainly accumulated in the gastrointestinal tract suggesting that gastrointestinal metabolism was the main pathway of SF in super early metabolism. In addition, a small proportion of SF was excreted through the urinary system over time. Meanwhile, as the metabolic organ, the traced SF in liver kept at a low level in the early, similar to that of the background. Detailed dynamic changes of early *in vivo* distribution in interested organs were shown in Figure S1.

#### Primary in vivo distribution

SPECT images of the typical *in vivo* biodistribution of ^125^I-SF with four particle sizes from 1 h to 72 h were shown in Fig. 7B. The three kinds of SF (i.e., SF_200_, SF_200-450_, SF_450-800_) shared a similar* in vivo* distribution and degradation process, however, the rate of intestinal deposition and clearance were significantly influenced by the increase of SF particle size. In the images of SF_200_ clearance, the gastrointestinal tract was still the main organ where SF was deposited. Compared with early distribution, the amount of SF in the gastrointestinal tract showed a fluctuating and gradually downward trend, while rarely amounts of ^125^I-SF were concentrated in the bladder. At 72 h, a little free ^125^I from the iodinated SF accumulated in the thyroid. Similarly, for SF_200-450_ SF_450-800_, decreasing trends of deposition in the gastrointestinal system appeared after 10 h and little SF were observed in bladder. Particularly, the amount of SF with these three sizes in liver were maintained stable low level until 72 h.

Notably, SF_800_ was of totally different distributional characteristics from the other three particle sizes. As shown in Fig. 7B, SF_800_ was mainly distributed in the liver after 10 h and maintained until 72 h instead of gastrointestinal system, indicating SF with lager size was likely to returned to liver via enterohepatic circulation for further metabolism. In addition, thyroid was faintly visualized after 48 h.

## Discussion

In this study, the integrated diagnostic nuclide I-131 was used to label SFMS, demonstrating a good stability of nuclide labeling. Nuclear medicine imaging technology was used to conduct real-time localization and semi-quantitative monitoring of embolization microspheres, and to evaluate the accumulation of radioactive microspheres in tumor areas and non-tumor sites. After TARE treatment on rat liver cancer model, the tumor showed significant embolism efficacy, as tumor activity was significantly inhibited, but the normal tissue was not influenced. Based on the small animal tumor model, the feasibility of using nuclide labeled SFMS for TARE of liver cancer was preliminarily verified, which further provided the experimental basis for the functionalization and clinical transformation of SFMS. There are many recognized barriers of traditional embolic materials in clinical application, such as single function, fast drug release kinetics, insufficient X-ray absorption and embolic agent reflux [18]. To solve the problems above, the effective carrier of chemotherapeutic drug doxorubicin and radiotherapy sensitizer gold nanoparticles was developed by rational utilization of the properties of the porous structure and rich amino acid components of SF in this study. Based on the promising *in vitro* results of drug release and radiosensitization, this study focused on the therapeutic process and effect of selective internal radiotherapy with SFMS labeled with I-131 in rat hepatoma models. As an important branch of TAE technique, TARE has shown significant advantages in the treatment of liver cancer. TARE delivers therapeutic radiopharmaceuticals to tumors via arteries, simultaneously achieving embolization of artery inducing ischemia and necrosis of the tumor, and radiation killing of the tumor cells. In particular, unlike external radiotherapy, intra-radionuclide radiation accumulates therapeutic radiation dose at the tumor site through continuous irradiation of therapeutic beta or alpha radionuclide, resulting in significantly reduced side effects in patients. As shown in Fig. 4 that exhibits the long-term tracking of embolic agents in vivo, the advantages of fibroin-based TARE are mainly reflected in the significant reduction or even elimination of radiation to non-target organs and tissues.

In the perspective of embolic agent preparation, for most embolic materials, radionuclides cannot be directly labeled. When compared with those microspheres available in clinic, such as ThereSphere and SIR-Sphere, the biodegradablity guarantees the possibility of second and even multiple embolization; SF contains more than 5000 amino acids [19], which contains a large number of active amino acids, among which the molar percentage of tyrosine reaches 5.3%, becoming one of the most abundant active amino acids. The abundant side tyrosine provides a large number of active sites for radioactive iodine labeling. In the view of nuclides-loading, the abundant tyrosine contributed to the capacity of radioactivity to as high as 1924 ± 210 MBq/mg, meanwhile, the iodination was totally different with physical absorption or package in in vivo performance. In light of the unique biological properties of SF, this study developed the dual effects of protein microspheres on chemical labeling and physical adsorption of nuclides. No delabeling of nuclides was observed during the embolization period (until 160 h post embolization), resulting the killing effect of microsphere-based long-lasting embolization was realized. The undesirable separation of radioactive drugs and microspheres is another common problem in the clinical use of TARE. For example, yttrium-90 (^90^Y) radioactive microspheres made of resin or ceramic carrier in clinical use are easy to leak into the blood and cause serious adverse reactions such as myelosuppression and pulmonary fibrosis [20, 21]. In addition, non-degradable radioactive embolization microspheres (e.g., glass microspheres) are prone to ectopic embolization, resulting in potentially serious sequelae. Long-term SPECT imaging monitoring results confirmed that the SF, as a degradable microsphere, is easy to be degraded by protease and cleared within a few days (as shown in Fig. 7), thus effectively avoiding the potential side effects mentioned above.

In the perspective of the actual efficacy of TAE in the treatment of liver cancer, on the one hand, the most commonly used TAE materials are non-degradable embolizers, which achieves permanent embolization by blocking blood flow for a long period of time. However, the hypoxic environment of tumor induced by hepatic artery embolization is not conducive to the effect of therapeutic drugs and may stimulate the up-regulation of vascular growth factors such as VEGF, promoting the growth and metastasis of liver cancer cells. As a result, the overall effective rate and long-term efficacy of TAE are unsatisfactory. On the other hand, single-function embolic agents often fail to meet complex clinical needs, such as lacking imaging or drug-carrying capabilities, resulting in difficult postoperative evaluation and poor therapeutic outcomes. The aforementioned issues highlight the necessity of developing novel and more efficient embolic agents, capable of addressing these shortcomings. In recent years, the development of biodegradable embolic agents and controlled drug release have made significant progress. The SF used in this study is a biodegradable embolic agent. Not only is SF non-toxic and non-immunogenic, but its degradation cycle is completed within a few weeks and the degradation products are non-toxic and harmless to the organism [22]. These characteristics of SF make the interventional embolization operation highly reproducible[23, 24]. The results of SEM in this study also proved that SFMS were of good pellet formation, regular morphology and no obvious adhesion between microspheres, which was conducive to smooth injection of SFMS into blood vessels through the catheter. In this study, the most commonly used radionuclides ^125^I and ^131^I were used to label SFMS by Iodogen method, and the labeling rates were all higher than 99%, and the good stability of radioactive SFMS could meet the requirements of SPECT imaging and TARE therapy. Previous studies have shown that SFMS can be chemically modified to achieve ideal drug adsorption and slow release [25, 26]. Based on the large number of pore structures on the surface and inside of SFMS, the microspheres can effectively load doxorubicin and AuNPs by a simple blending method, thus achieving slow drug release and increasing the dose deposition of ^131^I rays in the tumor area and producing high efficiency of radiation effect. Further, AuNPs after ionization excitation can produce a large number of free radicals and secondary electrons deposited in tumor tissue, causing DNA damage leading to tumor cell death, so as to achieve tumor sensitization effect of radiotherapy [27]. AuNPs-loaded microspheres synthesized in this study sensitized in situ radiotherapy of ^131^I-labeled SF, providing a functional carrier for the microspheres to enhance selective internal radiotherapy. In the drug design of SFMS and SF-based carriers, the particle size, may affecting drug metabolism and application, is also an important factor that should be considered [28]. For example, according to modifying the dendrimer size, ployamidoamine-based gadolinium complex contrast agents will alter the route of excretion [29–31]. Similar to nano and sub-micron materials, particle size of SF may potentially influence its blood clearance, metabolic pathways, and metabolic rate, especially when SF takes spherical shape in biomedical applications. Hence, as a biodegradable embolic material, the *in vivo* metabolic pathway of SF after it is decomposed into fragments under the action of various biological active enzymes *in vivo* is worthy of attention. Taking these features proved in Fig. 7, smaller sized contrast agents excreted through the kidney are suitable as functional renal contrast agents, while larger sized are found better suited for blood and lymphatic imaging. The similar phenomenon of size effect also exists in gold and iron oxide nanoparticles [32, 33]. Hence, figuring out the corresponding particle size effect on biological behaviors is useful for effective and purposeful drug design.

## Conclusions

As a bio-safe and degradable embolic material, SF provides sufficient and stable loading space for drugs. The rich amino acid types provide convenience for the labeling of various nuclides. The distribution and embolization effect of radioactive embolization microspheres can be observed in real time during the operation after the integration of diagnosis and treatment of nuclides. Therefore, SFMS have the potential to be transformed into functional embolization microspheres, providing the choice of embolization materials for the treatment of liver cancer and liver metastasis based on TARE.

### Electronic supplementary material

Below is the link to the electronic supplementary material.

Supplementary Material 1

## Data Availability

The data of this study is available from the corresponding authors on reasonable request.

## References

[1] Bray F, Ferlay J, Soerjomataram I, Siegel RL, Torre LA, Jemal A. Global cancer statistics 2018: GLOBOCAN estimates of incidence and mortality worldwide for 36 cancers in 185 countries. CA: a Cancer Journal for Clinicians. 2018;68:394–424. 10.3322/caac.21492 30207593

[2] Tsochatzis EA, Germani G, Burroughs AK (2010). Transarterial chemoembolization, transarterial chemotherapy, and intra-arterial chemotherapy for hepatocellular carcinoma treatment. Semin Oncol.

[3] Negussie AH, Dreher MR, Johnson CG, Tang Y, Lewis AL, Storm G (2015). Synthesis and characterization of image-able polyvinyl alcohol microspheres for image-guided chemoembolization. J Mater Sci: Mater Med.

[4] Wei-Ze L, Wen-Xia H, Ning Z, Shu-Miao H, Fei L, Li-Na F (2020). A novel embolic microspheres with micro nano binary progressive structure for transarterial chemoembolization applications. Eur J Pharm Sci.

[5] Ni HC, Yu CY, Chen SJ, Chen LC, Lin CH, Lee WC (2015). Preparation and imaging of rhenium-188 labeled human serum albumin microsphere in orthotopic hepatoma rats. Appl Radiat Isot.

[6] Meyer C, Pieper CC, Ezziddin S, Wilhelm KE, Schild HH, Ahmadzadehfar H (2014). Feasibility of temporary protective embolization of normal liver tissue using degradable starch microspheres during radioembolization of liver tumours. Eur J Nucl Med Mol Imaging.

[7] Chen M, Shu G, Lv X, Xu X, Lu C, Qiao E (2022). HIF-2α-targeted interventional chemoembolization multifunctional microspheres for effective elimination of hepatocellular carcinoma. Biomaterials.

[8] Tischfield DJ, Gurevich A, Johnson O, Gatmaytan I, Nadolski GJ, Soulen MC (2022). Transarterial embolization modulates the immune response within target and nontarget hepatocellular carcinomas in a rat model. Radiology.

[9] Omenetto FG, Kaplan DL (2010). New opportunities for an ancient material. Science.

[10] Hong H, Lee OJ, Lee YJ, Lee JS, Ajiteru O, Lee H (2020). Cytocompatibility of modified silk fibroin with glycidyl methacrylate for tissue engineering and biomedical applications. Biomolecules.

[11] Gholipourmalekabadi M, Mozafari M, Gholipourmalekabadi M, Nazm Bojnordi M, Hashemi-soteh MB, Salimi M et al. In vitro and in vivo evaluations of three-dimensional hydroxyapatite/silk fibroin nanocomposite scaffolds: three-dimensional HAp/SF nanocomposite scaffolds. Biotechnology and Applied Biochemistry. 2015;62:441 – 50. 10.1002/bab.1285 25196187

[12] Kundu B, Rajkhowa R, Kundu SC, Wang X (2013). Silk fibroin biomaterials for tissue regenerations. Adv Drug Deliv Rev.

[13] Gong H, Wang J, Zhang J, Wu J, Zheng Z, Xie X (2019). Control of octreotide release from silk fibroin microspheres. Mater Sci Engineering: C.

[14] Gholipourmalekabadi M, Sapru S, Samadikuchaksaraei A, Reis RL, Kaplan DL, Kundu SC (2020). Silk fibroin for skin injury repair: where do things stand?. Adv Drug Deliv Rev.

[15] Xu X, Wang X, Qin C, Khan A, ur R, Zhang W, Mo X (2021). Silk fibroin/poly-(L-lactide-co-caprolactone) nanofiber scaffolds loaded with Huangbai Liniment to accelerate diabetic wound healing. Colloids Surf B.

[16] Niemiec SM, Louiselle AE, Hilton SA, Dewberry LC, Zhang L, Azeltine M (2020). Nanosilk increases the strength of diabetic skin and delivers CNP-miR146a to improve wound healing. Front Immunol.

[17] Stabin MG, Siegel JA (2003). Physical models and dose factors for use in internal dose assessment. Health Phys.

[18] Hu J, Albadawi H, Chong BW, Deipolyi AR, Sheth RA, Khademhosseini A (2019). Advances in biomaterials and technologies for vascular embolization. Adv Mater.

[19] Murphy AR, Kaplan DL (2009). Biomedical applications of chemically-modified silk fibroin. J Mater Chem.

[20] Salem R, Mazzaferro V, Sangro B (2013). Yttrium 90 radioembolization for the treatment of hepatocellular carcinoma: Biological lessons, current challenges, and clinical perspectives: Hepatology. Hepatology.

[21] Giunchedi P, Maestri M, Gavini E, Dionigi P, Rassu G. Transarterial chemoembolization of hepatocellular carcinoma. Agents and drugs: an overview. Part 1. Expert opinion on drug delivery. 2013;10:679–90. 10.1517/17425247.2013.770733 23406440

[22] Liu B, Song Y, Jin L, Wang Z, Pu D, Lin S (2015). Silk structure and degradation. Colloids Surf B.

[23] Weng L, Rostamzadeh P, Nooryshokry N, Le HC, Golzarian J (2013). In vitro and in vivo evaluation of biodegradable embolic microspheres with tunable anticancer drug release. Acta Biomater.

[24] Niessen C, Unterpaintner E, Goessmann H, Schlitt HJ, Mueller-Schilling M, Wohlgemuth WA (2014). Degradable starch microspheres versus ethiodol and doxorubicin in transarterial chemoembolization of hepatocellular carcinoma. J Vasc Interv Radiol.

[25] Leong W, Kremer A, Wang D-A (2016). Development of size-customized hepatocarcinoma spheroids as a potential drug testing platform using a sacrificial gelatin microsphere system. Mater Sci Engineering: C.

[26] Pritchard EM, Valentin T, Panilaitis B, Omenetto F, Kaplan DL (2013). Antibiotic-releasing Silk Biomaterials for infection Prevention and Treatment. Adv Funct Mater.

[27] Sun G, Wang T, Li X, Li D, Peng Y, Wang X (2018). Sub-micrometer Au@PDA-^125^I particles as theranostic embolism beads for radiosensitization and SPECT/CT monitoring. Adv Healthc Mater.

[28] Li X, Hu Y, Xiao J, Cheng D, Xiu Y, Shi H (2015). Morphological effect of non-targeted biomolecule-modified MNPs on reticuloendothelial system. Nanoscale Res Lett.

[29] Wang B, Sun Y, Davis TP, Ke PC, Wu Y, Ding F (2018). Understanding effects of PAMAM dendrimer size and surface chemistry on serum protein binding with discrete molecular dynamics simulations. ACS Sustainable Chem Eng.

[30] Gao Y, Wang J, Chai M, Li X, Deng Y, Jin Q (2020). Size and charge adaptive clustered nanoparticles targeting the Biofilm Microenvironment for chronic lung infection management. ACS Nano.

[31] Sheikh S, Nasseri MA, Chahkandi M, Allahresani A, Reiser O (2020). Functionalized magnetic PAMAM dendrimer as an efficient nanocatalyst for a new synthetic strategy of xanthene pigments. J Hazard Mater.

[32] Engstrom AM, Faase RA, Marquart GW, Baio JE, Mackiewicz MR, Harper SL (2020). Size-dependent interactions of lipid-coated gold nanoparticles: developing a better mechanistic understanding through model cell membranes and in vivo toxicity. IJN.

[33] Yang L, Kuang H, Zhang W, Aguilar ZP, Xiong Y, Lai W (2015). Size dependent biodistribution and toxicokinetics of iron oxide magnetic nanoparticles in mice. Nanoscale.

