# Supplementary material for: Silk fibroin-based embolic agent for transhepatic artery embolization with multiple therapeutic potentials
Source: J Nanobiotechnology. 2023 Aug 19;21:278. doi: 10.1186/s12951-023-02032-9 (PMC10439629; doi:10.1186/s12951-023-02032-9)
Supplement: Supplementary file 1 — Supplementary Material 1 [file 12951_2023_2032_MOESM1_ESM.doc]

**
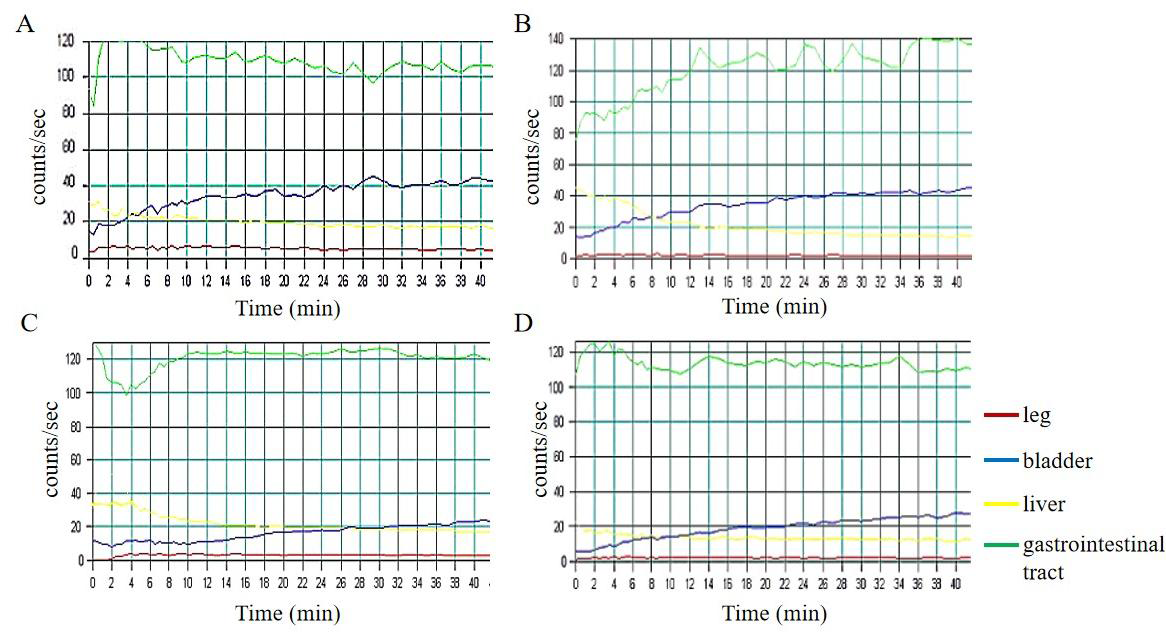
**

**Figure S1** The dynamic change curve of radioactivity counts in each interested organ (liver, gastrointestinal tract and bladder) in the first 40 min after injection of ^125^I-SF via rat tail vein. (A) SF_200_, (B) SF_200-450_, (C) SF_450-800_, (D) SF_800_.


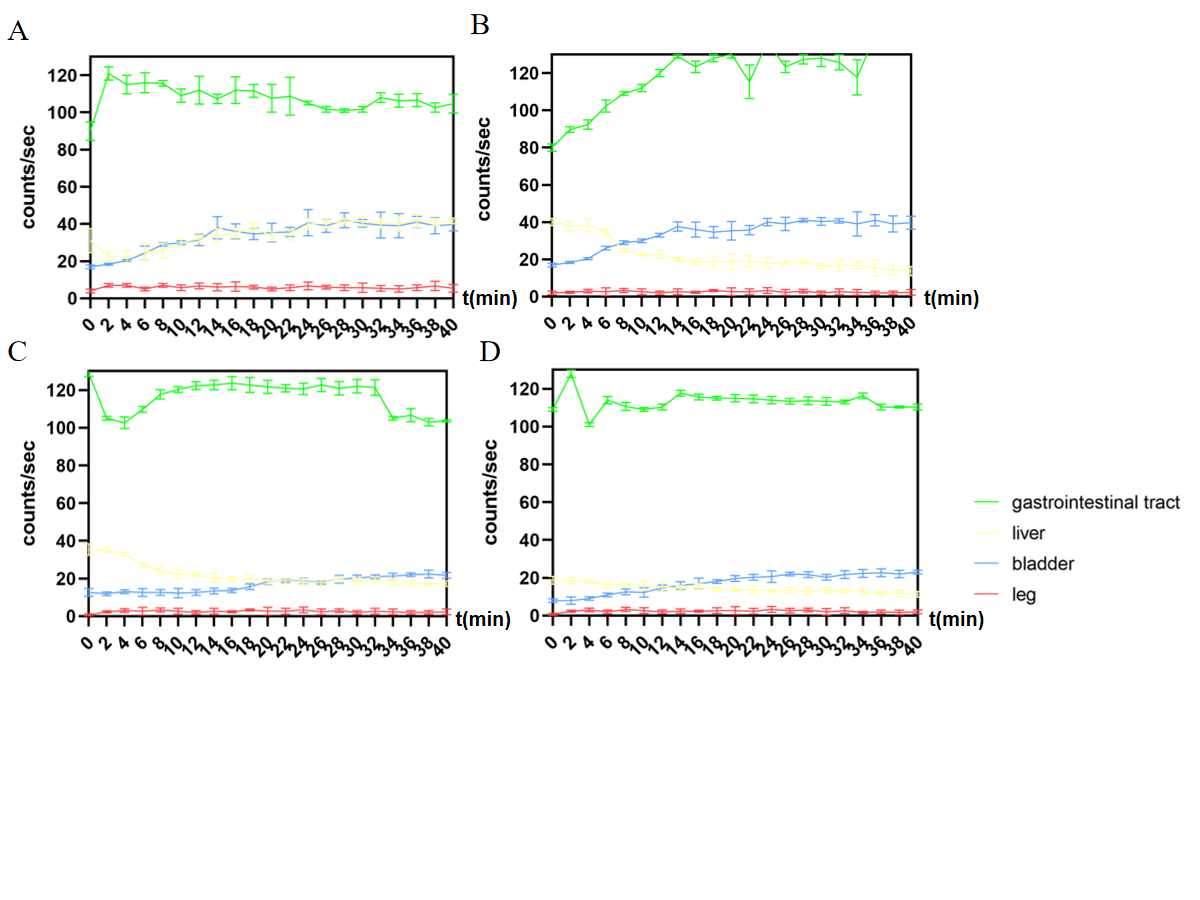


**Figure S2** The change trend of radioactivity counts in each interested organ (liver, gastrointestinal tract and bladder) in the first 40 min after injection of ^125^I-SF via rat tail vein. (A) SF_200_, (B) SF_200-450_, (C) SF_450-800_, (D) SF_800_.
